# Approaches for Modifying Oxide-Semiconductor Materials to Increase the Efficiency of Photocatalytic Water Splitting

**DOI:** 10.3390/ma15144915

**Published:** 2022-07-14

**Authors:** Svetlana Grushevskaya, Irina Belyanskaya, Oleg Kozaderov

**Affiliations:** Department of Physical Chemistry, Faculty of Chemistry, Voronezh State University, 1 Universitetskaya pl., 394018 Voronezh, Russia; belyanskaya_98@mail.ru (I.B.); kozaderov@vsu.ru (O.K.)

**Keywords:** photocatalytic water splitting, photoelectrochemical water splitting, hydrogen production, oxide semiconductor, nanostructure, heterojunction, doping, modification, sensitizing

## Abstract

The constant increase in the amount of energy consumed and environmental problems associated with the use of fossil fuels determine the relevance of the search for alternative and renewable energy sources. One of these is hydrogen gas, which can be produced by sunlight-driven photocatalytic water splitting. The decisive role in the efficiency of the process is played by the properties of the photocatalyst. Oxide materials are widely used as photocatalysts due to their appropriate band structure, high-enough photochemical stability and corrosion resistance. However, the bandgap, crystallinity and the surface morphology of oxide materials are subject to improvement. Apart from the properties of the photocatalyst, the parameters of the process influence the hydrogen-production efficiency. This paper outlines the key ways to improve the characteristics of oxide-semiconductor photocatalysts with the optimum parameters of photocatalytic water splitting.

## 1. Introduction

The growing consumption of fossil fuels induces environmental problems, of which the main ones are air pollution and global warming [1,2,3,4,5,6]. Hence, the development of alternative and renewable energy sources to replace traditional energy sources is a priority task. One of the most promising renewable fuels is hydrogen gas, which can be obtained by sunlight-driven photocatalytic water splitting. The possibility of such a transformation in a photoelectrochemical cell with a TiO_2_ photoanode was demonstrated for the first time by Honda and Fujishima [7].

The advantage of using hydrogen is that the only byproduct is water [5,6,8], regardless of whether the hydrogen was burned or reacted in an electrochemical cell. On the other hand, water is one of the most common, accessible and practically unlimited substances/sources of hydrogen [3]. The advantage of using sunlight to split water is that the amount of potentially available solar energy reaching the surface of the Earth is about 8.6 × 10^4^ TW per year, which far exceeds the total human energy consumption (about 19 TW in 2018) [4]. At the same time, the share of visible light (400–800 nm) in the solar spectrum is close to 50%. Therefore, it is important to use the solar spectrum as efficiently as possible for large-scale hydrogen production by photocatalytic water splitting. The solution of this problem includes the choice of photocatalyst material with appropriate band structure, tunable morphology and crystallinity.

Different photocatalysts are reported in the literature such as oxides, phosphides, sulfides, chalcogenides, transition metal carbides, carbon-based nanomaterials and other metal-free catalysts [9]. Metal oxides have several important characteristics for photocatalytic water splitting compared to other semiconductor materials. Their main advantages are earth abundance and a relatively large bandgap that results in high photovoltage, required for water splitting. Metal oxides are characterized by stability in aqueous media compared to non-oxide semiconductors. In addition, well-established low-cost routes can prepare a variety of oxide semiconductors with a wide range of optoelectronic properties [10]. For these reasons, oxide semiconductors are considered as promising materials for photocatalytic water splitting [4,5,6,8,10,11,12,13]. However, only some of them have been tested with remarkable success, as there are several criteria to be met simultaneously: corrosion potentials less favorable than water-splitting half-reactions; proper band alignment for large photovoltage generation; and favorable band position for the desired redox reaction. To date, no oxide-semiconductor photocatalyst with the entire set of parameters for efficient water splitting has been found so far. Therefore, the aim of modern research is to find the most effective strategies for oxide-catalyst modification.

This review critically discusses the different approaches to modify the oxide photocatalysts and to increase their efficiency in water splitting, including doping with metal or nonmetal ions, sensitization with quantum dots, modification of the crystal structure, improvement of crystallinity, nanostructuring, the use of heterojunctions and cocatalysts as well as the promotion of surface passivation in order to reduce corrosion.

It should be considered that along with hydrogen production, oxygen is also generated in this reaction. The rate of the latter four-electron process is lower than the rate of two-electron hydrogen production and must also be catalyzed. In this regard, a great attention is paid to the intensification of the oxygen evolution reaction [3].

Along with the properties of oxide-semiconductor photocatalytic materials, the composition of the solution, pH level, light intensity, temperature, applied potential, i.e., main parameters influencing the efficiency of water splitting are considered in this review.

Thus, the purpose of this review is to reveal the approaches to increase efficiency of the modified oxide-semiconductor materials and to optimize parameters of the photocatalytic water splitting with their use.

## 2. Mechanism of Photocatalytic Water Splitting

In order to choose an appropriate oxide semiconductor photocatalytic material, strategies of its modification, and optimum parameters for increasing hydrogen production, it is necessary to examine the mechanism of the photocatalytic water splitting and the role of the oxide photocatalyst in this process.

### 2.1. The Main Stages of the Photocatalytic Water Splitting

Photocatalysis includes four main stages [11,14]:

– the absorption of light by a semiconductor;
– excitation of charge carriers;
– separation and transfer of charge carriers;
– surface catalytic reactions.

The first stage is the irradiation of a semiconductor material with an energy higher or equal to its bandgap.

The absorption of light results in the second stage, which is the photogeneration of the electrons e^−^ and holes h^+^:

Catalyst → Catalyst (e^−^ + h^+^). (1)

In case of a wide-bandgap semiconductor, the electrons and holes can be generated only in the high-energy ultraviolet (UV) region of the solar spectrum. In a semiconductor with donor- or acceptor-energy levels the bandgap is narrowed. Therefore, in such semiconductors the photogeneration of nonequilibrium charge carriers becomes possible even under visible-light illumination.

The next stage is the separation of electrons and holes between the valence band (VB) and conduction band (CB). The photogenerated electrons transfer to the CB while the photogenerated holes stay in the VB.

Finally, charge carriers take part in surface catalytic reaction. Photogenerated electrons participate in the multistage process of proton reduction to molecular hydrogen:

2H^+^ + 2e^−^ → H_2_. (2)

In Process (2), the initial stage, namely the proton adsorption on the active centers of the semiconductor surface, is crucial.

Photogenerated holes are also involved in the reactions. Being powerful oxidizing agents, they can oxidize water and organic substances, such as alcohols:

H_2_O + 2h^+^ → 1/2O_2_ + 2H^+^, (3)

RCH_2_OH + 2h^+^ → RCHO + 2H^+^. (4)

### 2.2. The Main Conditions for Water Splitting

In addition to the presence of photoexcited charge carriers, the correct arrangement of band edges relative to Fermi levels of H_2_O/H_2_ and O_2_/H_2_O systems is necessary:

(5) $$E_{{F(O}_{2}{/H}_{2}O)}{=E}_{{F(O}_{2}{/H}_{2}O)\;}^{0}–\;kT\ln\left(\frac{a_{H^{+}}}{a_{H^{+}}^{0}}\right)–\frac{1}{4}kT\ln\left(\frac{P_{O_{2}}}{P_{O_{2}}^{0}}\right)\;,$$

(6) $$E_{{F(H}_{2}{O/H}_{2})}{=E}_{{F(H}_{2}{O/H}_{2})\;}^{0}–\;kT\ln\left(\frac{a_{H^{+}}}{a_{H^{+}}^{0}}\right)+\frac{1}{2}kT\ln\left(\frac{P_{H_{2}}}{P_{H_{2}}^{0}}\right).\;$$

Here, $E_{F(H_{2}O/H_{2})}^{0}$ and $E_{F(O_{2}/H_{2}O)}^{0}$ are the standard Fermi levels of H_2_O/H_2_ and O_2_/H_2_O systems, $a_{H^{+}}$ and $a_{H^{+}}^{0}$ are the activity and standard activity of protons in solution, *P* and *P*^0^ are pressure and standard pressure of gases.

**Figure 1:**
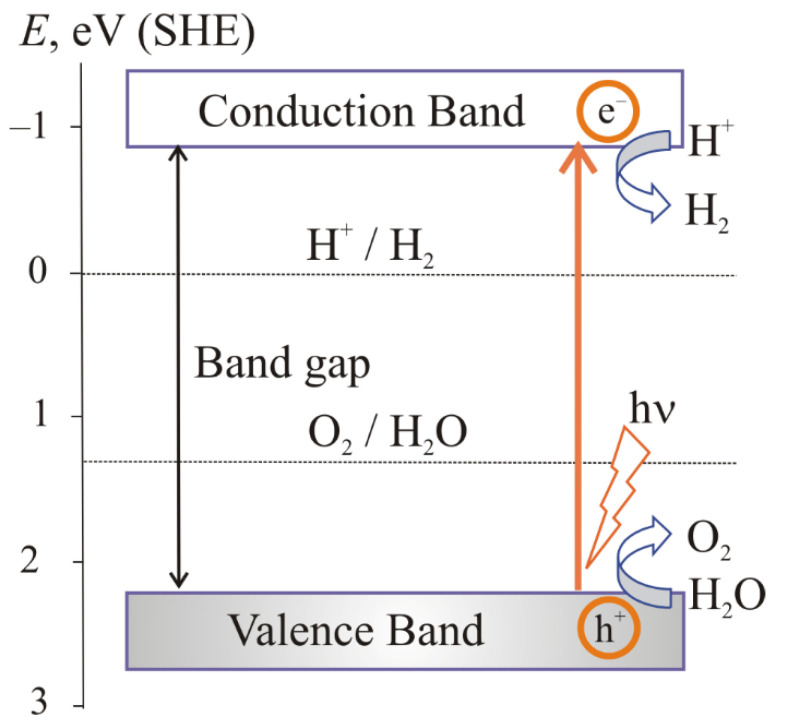
Generation of charge carriers in a semiconductor with band structure providing Processes (2) and (3) [6].

To provide the Reaction (2) the upper edge of the valence band must be more positive as compared to the Fermi levels of O_2_/H_2_O systems (5) (Figure 1). To provide the reaction (3) the lower edge of the conduction band must be more negative as compared to the Fermi levels of H_2_O(H^+^)/H_2_ systems (6) (Figure 1).

**Figure 2:**
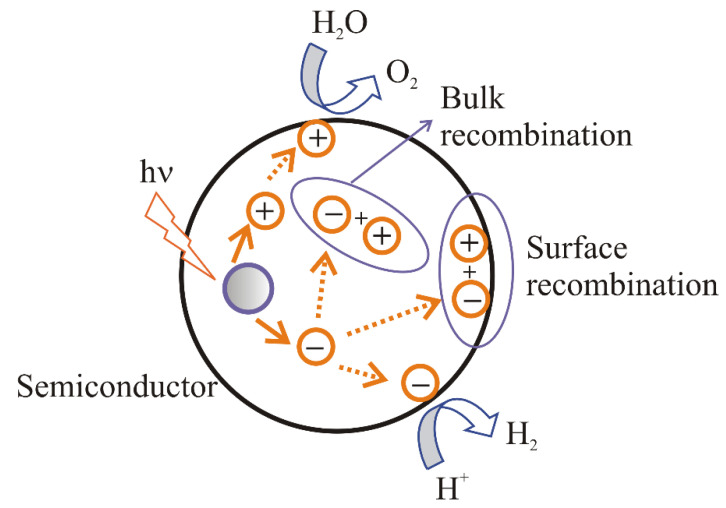
Photogeneration of charge carriers in a semiconductor (SC) and possible ways of their distribution [16].

The electric field formed at the interface as a result of achieving thermodynamic equilibrium provides the separation of photoexcited charge carriers [15]. The following ways for the distribution of electrons and holes are possible [11] (Figure 2):

(i). recombination of electrons and holes on the surface of a semiconductor;
(ii). recombination of electrons and holes in the bulk of a semiconductor;
(iii). transport of the electrons to the surface and participation in the reduction reaction;
(iv). transport of the holes to the surface and participation in the oxidation reaction.

In cases (a) and (b), Process (1) proceeds in the opposite direction, heat is released, and charge carriers are excluded from further operations. In cases (c) and (d), an electrochemical process is carried out.

The main problem that arises during photocatalytic water splitting is the irreversible recombination of charge carriers with the release of heat. To prevent the recombination, it is necessary to maintain the stability of the separation of charge carriers due to the electric field. For this purpose, photocatalytic water splitting is usually studied in electrolyte solutions (for example, Na_2_S and KI) in the presence of a sacrificial reagent [17,18,19,20,21]. Some examples of the sacrificial reagents are presented in Table 1.

The compounds of the electrolyte do not undergo reduction or oxidation by electrons and holes. However, they provide the transport of ions and electrons, and therefore increase the efficiency of photocatalytic water splitting [11].

The sacrificial substances can accept the holes [17] increasing the hydrogen formation efficiency because of more effective charge separation [22]. Moreover, the sacrificial substances can take part in electrochemical transformations. Alcohols are oxidized based on their standard oxidation potentials, which are lower than that of water [23], and they can participate in the processes of photoreforming into hydrogen and CO_2_ in the presence of H_2_O. The effectiveness of the most frequently used sacrificial agents with concentration of 20 vol.% increases in the following order: ethanol < methanol < ethylene glycerol < glycerol [17,19]. In [24], another row was observed for sacrificial agents with concentration of 1000 mg L^−1^: ethanol < glycerol < glucose < methanol. In accordance with [23], the efficiency of organic compounds depends on the number of hydroxyl groups, which defines the polarity, binding mode on the catalyst, adsorption strength and oxidation potential. In general, the higher the polarity of organic compound, the higher the efficiency of hydrogen evolution. Furthermore, hydrogen production is stimulated in the presence of α-hydrogen atoms in alcohols; therefore, ethanol with fewer carbon atoms attached to α-hydrogen atoms showed less efficient H_2_ formation as compared to other alcohols.

Apart from the type of sacrificial agent, its concentration influences the rate of the photocatalytic process. In [25], the increase in concentration of trimethylamine from 0.1 to 1.0 mol L^−1^ was observed to promote proper use of the catalyst active sites for improved mass transfer.

In general, the photocatalytic hydrogen generation increased by growing concentration of sacrificial agent because of its more diffusion [24]. However, this effect is limited by an optimal concentration of the sacrificial reagent. For example, for methanol the maximum efficiency of hydrogen production was observed in 5% solution; a further increase in the concentration does not lead to an increase in the amount of H_2_ due to the saturation of the photocatalyst surface [17].

The best way to choose an optimum sacrificial agent is to combine the high efficiency, low price, and possibilities of recycling. It is important to note that alcohols such as methanol and ethanol are rather valuable compounds in industry, so their consumption during H_2_ production can lead to unreasonable costs [26]. Other substances have also been tested including some renewable biomass, organic waste, or pollutants as electron donors, such as glycerol, triethanolamine, and glucose [27]. Their efficiency is less compared with methanol yet. However, utilizing the biomass-derived substances and waste organic materials as sacrificial agents can enhance photocatalytic hydrogen production reducing the costs of the technology, excluding use of valuable alcohols, and ensuring waste recycling.

### 2.3. The Mechanism of Photoelectrochemical Water Splitting

Photoelectrochemical (PEC) water splitting can be realized in a photoelectrochemical cell without sacrificial reagents. A voltage bias is an effective way to facilitate electrons and holes’ separation and their transfer during the PEC water-splitting process. [3]. A necessary condition is the excess of the minimum voltage for the decomposition of a compound [28]. For water, it is equal to the difference of potentials corresponding to the Fermi levels of H_2_O/H_2_ and O_2_/H_2_O systems (5) and (6). The theoretical voltage of water splitting for unity activities and pressures should be at least 1.23 eV:

(7) $$\Delta E=E_{F(H_{2}O/H_{2})}^{0}-E_{F(O_{2}/H_{2}O)}^{0}=1.23\;\mathrm{eV}.$$

Considering the overvoltage of partial processes (2) and (3), it can reach ~2.3 eV.

**Figure 3:**
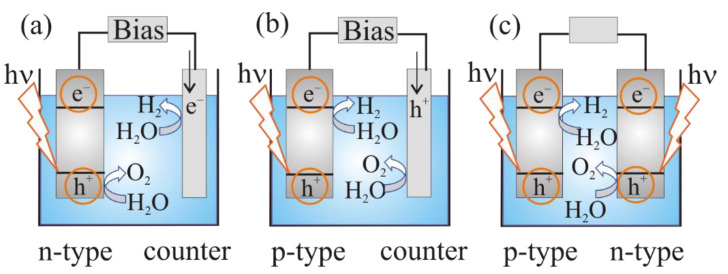
Photoelectrochemical cell for water splitting: (**a**) n-type semiconducting (SC) photoanode and Pt cathode; (**b**) p-type semiconducting (SC) photocathode and Pt anode; (**c**) semiconducting photoanode and photocathode (tandem system) [4].

The photoelectrochemical cell can be of three types:

– a photoanode (n-type semiconductor electrode) and a metal (platinum) cathode (Figure 3a);
– a photocathode (p-type semiconductor) and a metal (platinum) anode (Figure 3b):
– a photoanode (n-type semiconductor electrode) and a photocathode (p-type semiconductor (Figure 3c).

On the first step of the PEC water-splitting process charge carriers of semiconductor electrodes are photogenerated. The solar illumination causes the Fermi level rise in the n-type semiconductor bulk [28]. The flat-band potential *E*_fb_ corresponds to the maximum Fermi level in the photoelectrochemical cell. Hydrogen can be evolved at the counter electrode only if *E*_fb_ is above the Fermi level of H^+^/H_2_ system. Therefore, an external anodic bias is applied to increase the band bending in the semiconductor to maintain the required charge separation, and also to provide the overvoltage at the metal cathode, which is required to sustain the electric current flow. The further *E*_fb_ lies below the Fermi level of the H^+^/H_2_ system, the greater the bias [28].

After the electron–hole pairs appeared in the n-type photoanode, illuminated with photons with energies higher than the bandgap of the semiconductor, the minority charge carriers (holes) move to the interface and participate in oxidation reaction on the surface of the semiconductor photoanode. The main carriers (electrons) move into the bulk of the semiconductor, then through the external circuit to the counter electrode and participate in reduction reaction with the formation of hydrogen (Figure 3a).

The reduction in protons leads to the formation of hydrogen on a photocathode (p-type semiconductor), whereas on the counter electrode, H_2_O is oxidized (Figure 3b) [15,29]. In case of a cell constructed of n-type and p-type photoelectrodes (Figure 3c) the simultaneous reduction and oxidation of water molecules can be realized.

Economical hydrogen production requires an efficient interaction between the light, catalyst, and reagents [11]. The most important parameters by which the efficiency of photocatalytic water splitting is estimated include the stability of the photoelectrochemical cell and the rate of production of H_2_ or O_2_. Methods for the quantitative assessment of the main performance parameters of photocatalytic water splitting are discussed, for example, in [30]:

1. The turnover frequency (TOF) is the amount of hydrogen $n_{H_{2}}$, formed per unit of time *t* per gram of catalyst *m*_SC_:

(8) $$\mathrm{TOF}=\frac{n_{H_{2}}}{m_{\mathrm{SC}}t}.$$

2. The turnover number (TON) is the amount of hydrogen $n_{H_{2}}$ formed per mole of catalyst *n*_SC_:

(9) $$\;\mathrm{TON}=\frac{n_{H_{2}}}{n_{\mathrm{SC}}}.$$

3. The quantum yield (QY) is the ratio of the number of reacted electrons $n_{e^{-}}$, to the number of absorbed photons $n_{\mathrm{ph}}^{\mathrm{ads}}$:

(10) $$\mathrm{QY}(\%)=\frac{n_{e^{-}}}{n_{\mathrm{ph}}^{\mathrm{ads}}}\cdot 100\%\;.$$

4. Apparent quantum yield (AQY) is the ratio of the double amount (mol) of hydrogen $n_{H_{2}}$ to the number of emitted photons *n*_ph_:

(11) $$\mathrm{AQY}(\%)=\frac{{2n}_{H_{2}}}{n_{\mathrm{ph}}^{}}\cdot 100\%\;.$$

The minimum level for commercial application of photocatalytic water splitting is usually considered to be 10% quantum yield, 1000 h of stable operation and high rates of hydrogen and oxygen production [3,4,6,31,32,33]. For the achievement of such characteristics, the potential of the energy level at which holes are located in a semiconductor electrode must be at least 1.6 V [5], and the photocurrent density, measured in a photoelectrochemical cell under illumination, must not be less than 8.2 mA cm^−2^ [4]. The photocatalyst material plays a decisive role in the achievement of a high efficiency of water splitting.

## 3. Requirements for Photocatalysts

An ideal semiconductor electrode for photocatalytic water splitting should have the following properties [4]:

(i). Bandgap ensures the most effective light absorption of a wide spectrum;
(ii). The lower edge of the conduction band and the upper edge of the valence band are more negative than the hydrogen evolution potential, and more positive than the oxygen evolution potential, respectively;
(iii). A low number of defects for efficient charge transfer and reduced possibility of charge carriers’ recombination;
(iv). High corrosion resistance and photochemical stability;
(v). Low cost.

### 3.1. The Value of Bandgap

**Figure 4:**
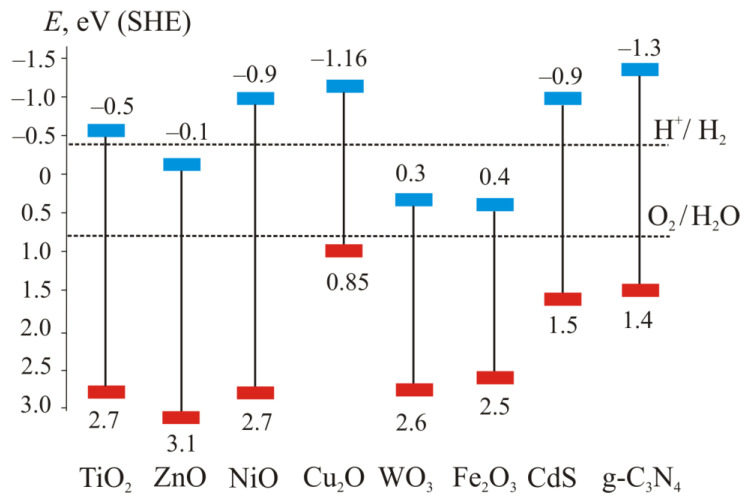
Band structure of some semiconductors and the Fermi levels of processes (2) and (3) at pH 7.

For highly efficient water splitting, a semiconductor photocatalyst should have a bandgap of more than 3 eV (equivalent to a wavelength of less than 414 nm) [6]. The classical examples are TiO_2_, ZnO and NiO (Figure 4). TiO_2_ is predominantly utilized as a photocatalyst because of its chemical stability, high reactivity and suitable band-edge positions, in addition to being known to be a cost-effective, nontoxic and biocompatible photocatalyst [34]. However, semiconductors with such a bandgap can only absorb ultraviolet light, which is only 4–5% of the entire solar spectrum. Therefore, it is advisable to use photocatalytic semiconductors with a smaller bandgap suitable for absorbing visible or near-IR light which are 42 and 40% of the solar spectrum, respectively.

From this point of view, such materials as metal oxides, chalcogenides, sulfides, phosphides and nonmetallic nitrides can be considered as perspective semiconductor photocatalysts [5,6,16,35,36]. Some chalcogenides also have fairly narrow bandgaps; however, they subject to photocorrosive degradation. Metal oxides are characterized by stability in aqueous media and low cost compared to non-oxide semiconductors. At the same time, the disadvantage of most oxide semiconductors is that the edge of the valence bands is determined by O2p orbitals, a potential of which is higher than 3.0 eV on the standard hydrogen electrode scale (SHE) [33].

For water splitting under visible light, photocatalysts must have narrow bandgaps, stability under photo irradiation, easy accessibility, nontoxicity, low price and suitability CB and VB for H_2_ generation using a single photocatalyst [11].

### 3.2. The Arrangement of Band Edges

Although the arrangement of band edges in TiO_2_ is suitable for water splitting, the bandgap is too large to effectively harvest visible light.

The bandgap of some oxides, such as Fe_2_O_3_ and WO_3_, is less than 3.0 eV, which provides the visible-light absorption. However, the lower edge of the conduction band in this case is above 0 eV (Figure 4), i.e., the reaction (2) is not possible. Such semiconductor oxides are doped with metal or nonmetal ions or combined with another appropriate material to achieve an optimum band structure.

Some nonoxide materials could be recommended as efficient photocatalysts because of appropriate band structure. For example, graphitic carbon nitride (g-C_3_N_4_) is a non-toxic, low cost, and metal-free semiconductor with a narrow bandgap (2.7 eV) with band edges providing reactions (2) and (3). Moreover, it has high photocorrosion resistance due to the strong covalent C-N bonds [11]. However, its application is limited by low specific surface area and high recombination rate of photogenerated charge carriers. The way to improve the separation of charge carriers due to synergic effect of Ag_2_O/g-C_3_N_4_ is reported in [11]. The hydrogen production with Ag_2_O/g-C_3_N_4_ was 274 times higher than that of pure g-C_3_N_4_.

The bandgap of CdS is even narrower than the bandgap of g-C_3_N_4_. That is why cadmium sulfide shows a relatively high response to visible light [36]. The photocatalytic performance of CdS can be improved by combination with other semiconductors. Thus, CdS-ZnS composites exhibit higher activity for the hydrogen evolution reaction as compared to the individual sulfides. An additional increase of photocatalytic activity is provided with the use of noble metal cocatalysts or noble metal-free cocatalysts because of its low cost, such as Ni and Ni_2_P. In [36] the mixed Cd-Zn sulfide catalysts prepared by a coprecipitation method with following heat treatment formed the active Zn_0.78_Cd_0.22_S phase. Its photocatalytic activity was enhanced with the deposition of 0.05 wt.% Ni or 0.25 wt.% Ni_2_P.

However, the additional corrosion-protection measures remain relevant for these materials. That is why the oxide materials are considered as better candidates for long-term hydrogen production.

Despite many years of searching for the most suitable photoelectrode material among the many studied probable binary metal-oxide semiconductors, it has not yet been found [4,37]. In this regard, more complex three-component semiconductor materials based on metal oxides are of interest. It is assumed that by combining the components of ternary systems in different ways and thereby varying the bandgap structure, one can find the most optimal photoelectrode material [38,39], which is characterized by a high efficiency of photocatalytic water splitting.

### 3.3. The Number of Defects

One of the structure-tuning method for the modification of semiconductor oxide materials is the introduction of defects with extra electrons or holes [4]. The introduction of oxygen vacancies can improve the photocatalytic activity and stability of metal oxide. Oxygen vacancies in TiO_2_, WO_3_ and Fe_2_O_3_ as typical intrinsic defects influence their electronic and optical properties via the manipulation of their donor density [4]. In the crystal lattice of TiO_2_ the oxygen vacancies are existed along with Ti^3+^. The higher the oxygen-vacancy concentration, the more defect states of Ti^3+^ ions are produced. The oxygen vacancies can trap and prolong the life of electrons, whereas the regular lattice of the oxygen atom was taken by electrons and local state was formed by oxygen vacancies and Ti^3+^. Then, the holes in the VB and the electrons in the CB of TiO_2_ were additionally generated. This strategy can be effective to increase the lifetime of the electron–hole pairs for enhanced H_2_ production [4]. On the other hand, a high concentration of defects in metal oxides can decrease their photocatalytic efficiency because they act as recombination centers for charge carriers. It was shown that the enhanced H_2_ production [34] with controllable thickness and concentration of O_2_ vacancies in TiO_2_ was due to a sufficient number of active sites and improved mass transfer due to the presence of vacancies and linker defects.

### 3.4. Corrosion Resistance and Photochemical Stability

In addition to a high photocatalytic activity, oxide material should have a high corrosion resistance. Photocorrosion is the main reason causing the poor stability of photocatalysts [16]. In order to investigate corrosion resistance and photochemical stability, a long-time experiment or a repeated experiment is necessary.

The efficiency of ZnO is strongly limited by not only a large bandgap that prevents visible-light absorption, but also chemical instability under both anodic and cathodic bias. It was demonstrated [40] that the cobalt oxide-phosphate cocatalyst photodeposited into ZnO rod arrays improves the photocurrent onset potential by 0.23 V. Furthermore, photodeposition was advantageous over electrodeposition, as it deposits the cocatalyst at locations with high photohole concentrations protecting the oxide material from photo corrosion.

As a p-type semiconductor, Cu_2_O acts as photocathode for water reduction. However, the material undergoes oxidative decomposition in air and anodic or cathodic photo corrosion forming CuO or Cu. A combination of 4 nm Al_2_O_3_ and 11 nm TiO_2_ layers significantly reduces Cu_2_O photocorrosion [40].

The main limitation of nonoxide semiconductors such as CdS and other sulfide, oxysulfide, oxynitride and nitride is their low stability, which is usually due to photocorrosion. When PdS is loaded on CdS, the hydrogen evolution activity is stable for over 25 h, and even longer than 100 h for a scale-up test [41]. The bulk and surface parameters of PdS-loaded CdS remain nearly unchanged after reaction for 25 h. In contrast, the activity of H_2_ production on Pt/CdS decreases after several hours. Therefore, PdS oxidation co-catalyst protects CdS from photooxidation removing the photogenerated holes. This might be general for most photocatalysts, where the semiconductors tend to be oxidized by the photogenerated holes. Problems associated with photocorrosion of CdS and other metal sulfides can be also overcome with the use of suitable sacrificial electron donors, such as S^2−^/SO_3_^2−^ ions [36].

## 4. Strategies of the Metal Oxide Photocatalyst Modification

In general, to increase the efficiency of hydrogen production in the process of photocatalytic water splitting, five strategies for the modification of an oxide-semiconductor material are considered:

(i). Modification of the crystal structure and morphology;
(ii). Doping of a semiconductor with metal and/or nonmetal ions;
(iii). Sensitization of semiconductors by quantum dots;
(iv). Formation of solid solutions or heterojunctions;
(v). Application of a cocatalyst.

### 4.1. Modification of the Crystal Structure and Morphology

It is known that the photocatalytic activity of conventional metal-oxide photocatalysts for water splitting depends on the crystallinity and morphology of the material, determined by the conditions of its preparation [42,43,44,45,46]. First, the efficiency of separation and transport of photoexcited charges is significantly affected by the crystallinity of the material, the presence of defects and distortions of the crystal structure. In addition, the surface area and size of particles, the structure of the surface and active reaction sites are important.

An increase in photocatalytic activity can be ensured by increasing the number of active centers as a result of surface development and an increase in material dispersion [43]. The smaller crystalline particle size of photocatalysts, the shorter transition distances of photoexcited charge carriers to the active reaction centers on the semiconductor surface, so the faster this transition and the lower the probability of the electrons and holes’ recombination [4,5].

#### 4.1.1. Modification of the Crystal Structure

The main factors determining the total collection efficiency of photoexcited charge carriers are the total lifetime of electrons and holes, the distance that they must pass, and the rate of their recombination, which is mainly determined by the electronic structure of the semiconductor. Modification methods associated with improving crystallinity, reducing the number of defects and increasing the concentration of charge carriers lead to an increase in conductivity and the rate of charge carrier transfer.

The main method for improving crystallinity is annealing in air. For example, a decrease in the concentration of intrinsic defects in ZnO nanowires after their annealing in air was reported in [6]. Additional treatment with hydrogen can reduce the probability of recombination due to the passivation of zinc vacancy traps. The combination of annealing and hydrogen treatment can be used for obtaining material without the recombination of donor–acceptor pairs.

At the same time, it was shown in [47] that the efficiency of the water-splitting reaction in highly defective samples is higher than in samples without defects. In particular, an oxygen vacancy can trap an electron, prolonging its lifetime. Thus, for nanostructured hematite photoanodes with lattice defects, a photocurrent density of 1.2 mA cm^−2^ was registered for water splitting at 1.6 V (SHE), which was 1.5 times higher than the value obtained for a sample of the same material, but without defects. Lattice defects introduced into the hematite nanostructure reduce the potential of flat bands from 0.63 to 0.58 V (SHE), increase the charge carrier density from 1.83 × 10^20^ up to 5.44 × 10^20^ cm^−3^ and reduce the potential for oxygen evolution from 0.9 to 0.8 V (SHE), therefore increasing the efficiency of hydrogen production.

High-enough photocurrent densities of 1.24 and 1.86 mA cm^−2^ at 1.23 V (SHE) for nanowires and nanocorals, respectively, made of tin-doped hematite, were reported in [48]. According to ultrafast spectroscopy, the increased photoactivity of these nanostructures is a consequence of improved electrical conductivity and increased surface area, rather than the suppression of electron–hole recombination.

Making nanogroove patterns and varying surface orientation of titanium dioxide with low-energy argon-ion irradiation and photoetching in sulfuric acid, the authors [49] have achieved an optimal ratio between high surface-defect density and bulk crystallinity, which ensures enhancing photon adsorption and facilitating separation and diffusion of electrons and holes, respectively. It was found that in a 1 M NaOH under AM 1.5 G simulated solar light illumination (100 mW cm^−2^) the photocurrent density of Ar-TiO_2_ is more than 500 folds higher compared to that of p-TiO_2_.

#### 4.1.2. Modification of Size and Morphology

The efficiency of practically all oxide-semiconductor photocatalytic materials is limited mainly by the fast recombination of electrons and holes, primarily due to the mismatch between the small diffusion length of charge carriers and the relatively large photon penetration depth. This problem can be solved by adjusting the structure of the photocatalyst in order to reduce the distance of charge carriers to reach the active center on the surface, on the one hand, and to increase the absorption of photons, on the other. Typical designs of photoelectrodes include mesoporous nanostructures [16,50], zero-dimensional (0D) nanoparticles [21], one-dimensional (1D) nanostructures (nanowires [51,52], nanotubes [53], nanorods [54]), two-dimensional (2D) nanosheets or thin films [55], and three-dimensional (3D) hierarchical nanostructures [56,57,58,59,60].

For example, it was shown [50] that the introduction of a conducting mesoporous nano-ATO (antimony-doped tin oxide) film between hematite and an FTO (fluorine-doped tin oxide) substrate significantly increased the absorption of light, effectively reduced the recombination of the electronic charge and thus increased the photocurrent density to 0.83 mA cm^−2^, which was 3 times higher than in a similar structure without an ATO film.

One- and two-dimensional nanostructures (nanowires, nanorods, nanotubes, and nanosheets) are characterized by faster charge separation and larger surface area for photocatalytic reactions compared to zero-dimensional nanoparticles [20]. It was shown that the light absorption and carrier collection fluxes can be separated with arrays of nanowires and nanotubes [61,62]. The presence of one electron-transfer pathway in a one-dimensional structure determines the possibility of free movement of charges along the length of the nanostructure [63].

One-dimensional structures showed the electron diffusion up to 200 folds faster than in nanoparticles [64]. For instance, TiO_2_ NWs were characterized with 20% rise of H_2_ production compared with NPs of the same chemical composition. In metal-doped systems of Cu-In_2_O_3_ nanorods/TiO_2_ nanowires, the H_2_ production and quantum yield were up to 4 folds higher compared with Cu-In_2_O_3_/TiO_2_ nanoparticles [20]. The Cu/TiO_2_ nanorods and CuO/TiO_2_ nanofibers also showed an enhanced hydrogen formation [65].

Although nanowires are characterized by an increased specific surface area, electron diffusion in them occurs faster. As a result, redox reactions proceed at higher rates, while the rate of the side recombination process, on the contrary, decreases. On the contrary, the developed transport network in nanoparticles promotes accelerated charge recombination, and therefore the photon productivity decreases. Obviously, the structure of the nanomaterial significantly affects the photoactivity of the material. The morphology of nanowires strongly depends on preparation methods and has a significant effect on hydrogen production, which can be increased using a 1D/1D heterojunction due to the synergistic effect upon visible-light irradiation [20].

Longer nanowires are characterized by more efficient photon absorption, which is confirmed by the example of TiO_2_ NWs [51]. Modification of the nanowire by atomic layer deposition of an epitaxial rutile layer contributed to a more efficient separation of electrons and holes, which ultimately led to an increase in the photocurrent density. In turn, the deposition of quantum-well anatase nanowires onto the TiO_2_ nanotube significantly increased the total area of the interface of the tubular structure [53].

For using 2D materials in highly efficient photocatalytic processes, the synthesis of 2D materials with a tunable number of layers, degree of crystallinity, and edge-surface morphologies are of utmost importance [66]. Two-dimensional thin film electrodes composed of BiVO_4_ and WO_3_ layers on FTO were investigated as heterojunction composite photoanodes with relatively high photoactivity [55]. The anode composed of four layers of WO_3_ and one upper layer of BiVO_4_, and showed the highest photocurrent density at 0.7 V with 74% increase compared with bare WO_3_, and more than 7 times greater compared with bare BiVO_4_. In this heterojunction composite, the electrons photogenerated in BiVO_4_ are transferred to WO_3_ layers with good charge transport characteristics ensuring the enhanced photoactivity.

TiO_2_ NPs/ Ti_3_AlC_2_ binary composites [23] were characterized by more efficient transfer and separation of the photogenerated electron–hole pair. The metallic behavior of Ti_3_AlC_2_ plays important role to trap electrons. H_2_ production was approximately ten folds higher compared to TiO_2_(microparticles)/Ti_3_AlC_2_. The matter is that the larger microparticles of TiO_2_ have longer diffusion lengths, and smaller interfacial contacts. The sacrificial agents playing the role of hole scavengers to convert photo-induced holes into H^+^ additionally enhances the H_2_ formation, which is more than 50 times greater with glycerol than in pure water [23].

Tree-like composites of ZnO nanowires, covered with titanium dioxide nanosheets with copper (II) oxide nanoneedles showed enhanced H_2_ production [67] because of simultaneous effects of ZnO NWs high electron mobility, TiO_2_ high photocatalytic activity and stability, and the CuO expanded light absorption region.

Sufficiently effective light absorption with almost zero transmission was found for highly porous 3D hierarchical nanostructures with ZnO NRs grown on nanofibers [57]. The increase in photoelectrochemical activity is explained by the large contact area of ZnO nanorods with the electrolyte and the conduction channels formed by ITO particles. Unfortunately, zinc oxide has a limited light absorption (only in the UV region), and to expand it, ZnO is doped with different materials to synthesize heterostructures.

One of the simple ways to obtain semiconductor nanostructures with a controlled composition and morphology (including nanowires, nanosheets, nanoplates, nanorods, nanoneedles) is electrochemical deposition. In [68], this method was applied for the synthesis of ZnO/Cu_2_O structures used as photoanodes in photocatalytic water splitting. The duration of Cu_2_O deposition on ZnO nanorods plays a key role in the surface structure, and hence light absorption. It was shown that a 20 min deposition of copper oxide led to the lowest (compared with other samples) value of the polarization resistance (25486 Ω cm^−2^) and the highest photocurrent density (0.64 mA cm^−2^ at 0.5 V vs. Ag/AgCl electrode).

In [69], ZnO microrods modified with Cu_2_O nanocrystals were obtained by chemical deposition in an aqueous solution. These structures exhibit a higher photocatalytic activity in the decolorization of rodamin B solutions compared to individual ZnO and Cu_2_O oxides. Electrons photoexcited in Cu_2_O transfer in ZnO with a lower level of the conduction band. Holes from the ZnO valence band pass into the Cu_2_O valence band, migrating to the interface with the solution. At the same time, ZnO microrods can act as a channel for electron transport. Due to the good crystallinity of ZnO microrods, electrons can be transferred over long distances at a fairly high speed and without the recombination with holes. Electrons transferred to the ZnO surface can be absorbed by dissolved oxygen, eventually forming hydrogen peroxide and a hydroxyl radical, which oxidize rhodamine B.

The narrow bandgap of copper oxides CuO and Cu_2_O (1.4 and 2.0 eV, respectively), values of the maximum theoretical photocurrent density (35 mA cm^−2^ and 15 mA cm^−2^, respectively [8,70]), position of the conduction bands, as well as availability, low cost, and nontoxicity [71], make it possible to classify them as promising photocatalytic materials [72], including those for large-scale use. The disadvantage is a rather high rate of recombination of electrons and holes [73], a relatively short electron-diffusion length (10–100 nm) [74], as well as a tendency to chemical degradation, since copper oxides can be reduced to metallic copper at water-splitting potentials [71,75,76].

The characteristics of copper oxides can be improved by adding other oxides. Considering that NiO is a p-type semiconductor with a high mobility and concentration of photoinduced holes, and therefore acts as an effective cocatalyst in the reaction of hydrogen evolution [74], it was proposed to combine [77] copper and nickel oxides by depositing a thin layer of NiO_x_ on CuO. The photocurrent density on the resulting binary material is –1.02 mA cm^−2^ at 0 V (SHE), which is slightly higher than for CuO (–0.92 mA cm^−2^). According to the proposed mechanism of electron transfer between CuO and NiO_x_, the generation of an electron–hole pair occurs as a result of the absorption of visible light by copper oxide. Part of the photoexcited electrons is captured by the oxyhydroxide NiOOH present in the NiOx layer, which is confirmed by X-ray photoelectron spectroscopy data. NiOOH is capable of being reduced to nickel metal, which catalyzes the hydrogen evolution reaction. In turn, the increase in the pH of the near-electrode layer caused by the release of H_2_ promotes the regeneration of metallic nickel into NiO_x_.

### 4.2. Doping of an Oxide Semiconductor with Metal and/or Nonmetal Ions

One of the most effective methods for modifying wide-gap semiconductors to create impurity energy sublevels in the bandgap and expand the region of the light absorption is doping [4,6,78,79,80]. It is necessary to consider that only the formation of a donor sublevel above the VB increases the efficiency of the photocatalyst in the visible-light region. It was found [80] that in case of doping by cations the bandgap of a semiconductor decreases with a decrease in the dopant size. At the same time, the recombination of photoexcited electrons and holes is promoted in presence of metal ions in a semiconductor, and the photocatalyst performance decreases.

It was reported [16] that the advanced ion implantation of TiO_2_ with metals (V, Cr, Mn, Fe, Ni) shifted the absorption band of the material toward the visible-light region. The doping ions Pt^4+^- and Ag^+^ in TiO_2_ nanoparticles improved photocatalytic activities under visible-light or UV irradiation due promoted charge separation. However, some metal-ion dopants (Ni^2+^, Rh^3+^, or Cr^3+^) decrease the photocatalytic activity of TiO_2_ acting as recombination sites for electron–hole pairs, whereas codoping by Ni^2+^, Rh^3+^ or Cr^3+^ together with Ta^5+^, Nb^5+^ or Sb^5+^ led to efficient O_2_ evolution from water [16]. Nonmetal-ion (C, N, S)-doped TiO_2_ shifted the valence-band edge upward and improved photocatalytic activities in the visible-light region.

The crystal lattice of a semiconductor can be doped by substituting an O-atom or a metal atom with a nonmetal, placing a nonmetal atom in the interstitial space, including the interstitial space near an oxygen vacancy. The greatest effect is achieved when the O-atom is substituted by a C- or N-atom. The matter is that the 2p electronic states of oxygen and carbon (or nitrogen) are mixed, and the semiconductor bandgap narrows due to the upward displacement of the edge of the valence band.

Doping of ZnO with nitrogen improved the photocatalytic activity due to the valence-band modification [6], which demonstrated the expansion of the absorption region to the region of visible light, and increased the photocurrent [81,82]. The latter effect resulted from the combined effect of increased roughness by ZnO nanostructuring, and bandgap narrowing due to nitrogen doping was reported in [83]. Codoping with different nonmetallic ions is also possible, which suggests the appearance of a synergistic effect. In [6], the photocurrent enhancement in N-ZnO system is also explained by the combination of increased surface roughness for facilitating light harvesting from ZnO nanotetrapod branching and the increased absorption spectrum due to the N-doping-induced bandgap narrowing of ZnO [6].

In addition to expanding the light-absorption region, dopants can act as trapping centers for photoexcited electrons, thereby increasing the efficiency of the separation of electron–hole pairs. The impurity level can prolong the lifetime of photoexcited electron-hole pairs, leading to an increase in photocatalytic activity [6]. On the other hand, donor or acceptor levels, being discrete, can serve as recombination centers for photoexcited electron–hole pairs, which leads to a decrease in photocatalytic activity. Therefore, the doping of metal is not an ideal method.

The morphology/size and doping effective combination was shown in [84] for a NiO/NaTaO_3_ system doped with La, Ca, Sr and Ba. The probability of the reactions of photogenerated electrons and holes with water molecules increased due to the smaller particle size of the NaTaO_3_:La crystal (0.1–0.7 μm) compared with the nondoped NaTaO_3_ crystal (2–3 μm). Doping has an additional effect of the ordered surface nanostep formation, which enhanced the H_2_ and O_2_ evolution sites’ separation and improved the photocatalytic activity.

The photo- and thermal stability of the doping species in oxide semiconductors determines the sustainability of their increased photocatalytic activity. Advanced characterization of the La-doped NaTaO_3_ showed its regenerative stability six times without a significant loss of its photocatalytic activity [85]. The La species are considered to be photostable because of inalterability in their structure and oxidation state in the host NaTaO_3_. The phase transformation and crystallite growth of titanium dioxide is inhibited by the nitrogen and La^3+^; this improves the thermal stability of TiO_2_, which increases with the increase in La^3+^ doping [86].

### 4.3. Sensitization of Oxide Semiconductors by Quantum Dots

Quantum dots—that is, nanoparticles with a pronounced discreteness of energy levels—are characterized by rather high extinction coefficients and a relatively large intrinsic dipole moment. By changing the quantum dot size, one can vary the bandgap. From this point of view, quantum dots are promising sensitizers for oxide semiconductors with a wide bandgap. The influence of the degree of limitation of quantum sizes in suspended nanocrystalline dots on their photocatalytic activity was established in [87]. The possibility of fine tuning the photocatalytic activity by controlling the particle size was shown. Thus, CdSe quantum dots with a diameter equal to or higher than 3 nm did not exhibit photoactivity. In the range from 2.25 to 3 nm, the photoactivity of particles increased with a decrease in their diameter. If the size was lower than 2.25 nm, then the photoactivity of the particles decreased with a decrease in their diameter. In [87], this was explained by a decrease in the adsorption of high-energy photons.

An increase in the photocurrent density to ~3.1 mA cm^−2^ at 1.2 V (SHE), due to improved absorption in the visible range after sensitization of zinc oxide with CdSe nanoparticles, is one of the examples of the effectiveness of the sensitization process provided in [88]. CdSe-sensitized ZnO nanorods exhibiting a photocurrent density of 2.48 mA cm^−2^ at 0 V (SHE) were reported in [89]. ZnO nanotubes sensitized with CdSe showed ~5 mA cm^−2^ at 0.35 V (SHE) [90]. An array of ZnO nanorods sensitized with CdS nanoparticles with a high degree of crystallinity exhibited photocurrent densities of 23.7 and 15.8 mA cm^−2^ when illuminated with sunlight and visible light, respectively, at a voltage of 0 V [91].

The doping of oxide semiconductor materials with more than one component was very effective. For example, ZnO nanowires sensitized together with CdS and CdSe [92] were characterized by a photocurrent density of 13.9 mA cm^−2^ at 0.6 V (SHE).

Arrays of ZnO NWs sensitized by CdS and CdSe quantum dots are characterized by similar photocurrent values of about 12 mA cm^−2^ at 0.6 V (SHE) [93]. A higher photocurrent density of 17.5 mA cm^−2^ at 0 V (SHE) is inherent in systems consisting of a 2D shell of a ZnO nanosheet and nanowire core, sensitized by the same quantum dots, generating a photocurrent [94]. A photocurrent value of 11 mA cm^−2^ at –0.5 V (SHE) was obtained for CdS and CdSe-sensitized heterostructures of ZnO nanowires grown on WO_x_ rod-like nanowhisker [95]. CdTe sensitization of ZnO nanowires led to a more than 2-fold higher maximum photoconversion efficiency than observed for the original ZnO nanowires [96]. The higher efficiency of the sensitized nanostructures can be explained by the extension of the light-absorption spectrum and improvement of electrons and holes’ separation at the quantum dot/ZnO interface.

It was reported [6] that InP is another effective sensitizer. The high photocurrent measured in multi-bandgap-sensitized ZnO nanorod photoelectrode arrays resulted from the extended light absorption of InP quantum dots. The photoelectrochemical activity of TiO_2_ nanotubes was improved by extending absorption into the visible spectrum after the electrodeposition of cuprous oxide Cu_2_O as quantum dots [6].

The passivation of quantum dots with organic ligands (for example, to increase solubility before their deposition) can lead to the appearance of a sufficiently large potential barrier. As a result, photoexcited electrons cannot leave the quantum dot, which will lead to a weakening of the interaction or its loss. Usually, bifunctional organic molecules, attached to a semiconductor at one end and to a quantum dot at the other end, are used. The choice of the structure of this organic molecule should be made considering the maximum possible electron transfer. In several cases, the sequential construction of “metal oxide-quantum dot” by chemical or electrochemical deposition is used, which allows for complete elimination of the use of organic ligands. Combined approaches for increasing the absorption spectrum of an electrochemical cell are also studied.

### 4.4. Formation of Solid Solutions or Heterojunctions

Compared to individual oxide photocatalytic material, which usually have a low enough possibility to change the electronic structure by doping, the hybrid or integrated semiconductor systems show significant advantages of promoting the separation of charges [34]. The advantages of these systems are associated with the combination of the higher CB of one semiconductor and the lower VB of the other in water-splitting reactions. If the photoexcited electrons in one semiconductor could recombine with the holes in the other one, then the more powerful excited electrons and holes can stay separated.

An additional way for varying the photocatalyst band structure is the formation of a solid solution consisting of wide- and narrow-bandgap semiconductors.

For instance, solid solution (GaN)_1−x_(ZnO)_x_ can be obtained as a combination of gallium nitride and zinc oxide. In [97,98,99], this system showed a reproducible acceleration of the water-splitting process in visible light. The bandgap of this system depends on the composition x. For example, it is equal to 2.53 eV for direct transitions at x ~ 0.55 [100]. However, the formation of solid solutions is a rather laborious and expensive method for obtaining a material that is not very stable in a liquid medium under light irradiation.

The creation of heterojunctions is considered as more appropriate strategy that combines the properties of each component to increase overall efficiency. The heterojunction can expand the range of light absorption, promote the separation of photoexcited electrons from holes, minimize their recombination, and thus significantly increase the efficiency of hydrogen production [6].

**Figure 5:**
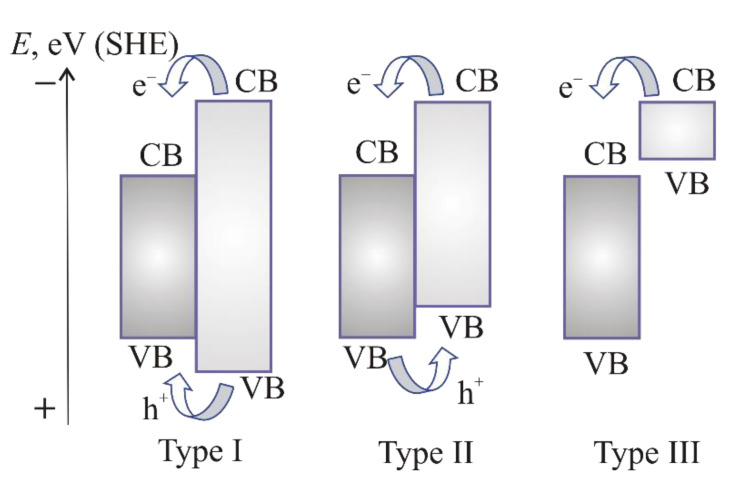
Three types of heterojunction depending on the transport mechanism of photoexcited charge carriers [13].

There are three main types of heterojunction depending on the band structure of semiconductors and transport mechanism of photoexcited charge carriers [11,13] (Figure 5).

A heterojunction structure of type I consists of a semiconductor B (SC B) with the valence (VB) and conduction (CB) bands located within the forbidden band of semiconductor A (SC A). The electron transfer from CB of semiconductor A to CB of semiconductor B is characterized by less negative potential (SHE). Moving of the holes is realized from the valence band of SC A to valence band of SC B.

A type II heterojunction is the combination of a semiconductor A with a more negative position of the CB and a semiconductor B with a less negative position of the CB. The valence band of SC A is less positive than the valence band of the SC B. In this heterojunction, the photoexcited electrons of SC A transfer in SC B, leaving photoexcited holes in the valence band of SC A. The electrons and holes move in opposite directions. Thus, the recombination of photoexcited charge carriers is excluded, and the photocatalytic properties are improved [11]. Reduction and oxidation reactions occur on different semiconductors. The redox reaction occurs at a lower voltage.

A type III heterojunction differs from a type II heterojunction by the mismatching of bandgaps caused by the tremendous staggered gap. Therefore, it is necessary to ensure a higher driving force for the transfer and separation of charge carriers [34].

An investigation of BiFeO_3_ (BFO), Bi_2_O_3_ films and a Bi_2_O_3_/BFO heterojunction (type II) obtained by pulsed laser deposition on ITO substrates showed [101] that the photocurrent of the Bi_2_O_3_/BFO heterojunction film was found to be twice as high compared to the BFO film, with the onset potential shifted in the positive direction by 240 mV.

A Z-scheme photocatalytic system was found to have sufficiently stronger redox ability and more effective separation of electrons and holes. The kinds of such scheme are as follows [11]:

– with shuttle redox mediator;
– with solid-state electronic linker;
– direct systems.

The first kind is a composition of two independent SCs with a redox pair of ions in the solution (Fe^3+^/Fe^2+^, IO_3_^−^/I^−^, NO_3_^−^/NO_2_^−^). These ions act as a mediator for electrons. Photoexcited holes in the VB of one semiconductor and photoexcited electrons in the CB of another semiconductor react with these ions during illumination with visible light. Electron donors accept holes from the VB of one semiconductor and electron acceptors consume electrons from CB of another semiconductor. This is a way to conserve holes in the CB of one semiconductor and electrons in the VB of another semiconductors. The conserved charge carriers can participate in redox reaction.

For a Z-scheme system with a solid-state electronic linker, the noble metals (Ag, Au) act as mediators for electrons. Electron transfer occurs through ohmic contact at the low-resistance solid interface. As a result, the fast recombination of photoinduced electrons in SC B and holes in SC A proceeds. Thus, a larger number of electrons and holes leave different active sites, which leads to an increase in the redox ability [102,103].

In the case of direct Z-scheme, semiconductor B has a low CB as compared to semiconductor A, and semiconductor A has a higher VB as compared to semiconductor B. Under light irradiations, photogenerated electrons in semiconductor B recombine at the heterojunction interface with the holes of semiconductor A [34].

In systems with a direct Z-scheme, the properties of the interface between two solid-state photocatalysts providing electron transfer are important. It was shown in [104] that the rate of hydrogen evolution from aqueous SO_3_^2−^ and S^2−^ solutions on heterojunctions with direct Z-scheme ZnO/Zn_0.2_Cd_0.8_S was 2.518 mmol h^−1^g^−1^. Such a material is rich in oxygen vacancies, and its conductivity is like that of a conductor. The rate of H_2_ formation for direct Z-scheme heterojunction ZnO/CdS was equal to 1.805 mmol h^−1^g^−1^, which was much higher than when using pure ZnO or CdS [105]. The authors concluded that the rate increased due to electrons moving from the conduction band of ZnO to the valence band of CdS.

The absorption spectrum of a photoanode can be expanded by depositing semiconductors with a smaller bandgap on the surface of a wide-gap semiconductor. The injection of photoexcited electrons into the conduction band of the basic oxides becomes possible, when the upper edge of the valence band of a wide-gap semiconductor is lower than the upper edge of the conduction band with a smaller bandgap. By varying the size, shape, and nature of the interaction between the particles of a semiconductor with a narrow bandgap, it is possible to change the energy of the system by modulating the energy levels. The combination of these approaches makes it possible to increase the efficiency of photocatalytic decomposition of water by doping the metal oxide with nonmetal atoms (for instance, nitrogen) and depositing a relatively narrow-gap semiconductor on its surface. Due to the synergistic effect, it is possible to shift the absorption spectrum to a large extent into the visible region.

The efficiency of multicomponent photocatalysts assembled from ZnO/Cu_2_O/CuO oxides according to the Z-scheme of water splitting was shown in [106]. First, ZnO particles were obtained by chemical precipitation; then, CuS was added. ZnO/CuS materials were thermally processed in a controlled oxygen atmosphere to form copper oxides. The maximum hydrogen production rate of 1.093 mmol g^−1^h^−1^ was obtained at 3% apparent quantum yield under standard solar irradiation for the optimum copper content (14.3%) in copper oxides.

### 4.5. Application of a Cocatalyst

Cocatalysts play a key role in water oxidation for both photocatalytic and PEC water splitting [41]. Since the process of water splitting involves surface chemical reactions, it is important to monitor the presence of active sites. Cocatalysts are usually loaded to introduce active sites for H_2_ evolution. If the active sites do not exist, then the photoexcited electrons and holes can recombine even under appropriate thermodynamic condition for the water splitting.

The role of the cocatalyst is to provide chemically active sites where the chemical reaction proceeds with lower activation barrier than on the oxide semiconductor. An additional function of cocatalyst nanoparticles is to extend the lifetime of charge carriers that reach the surface of the semiconductor by improving the electron–hole separation at the co-catalyst/semiconductor interface [107]. Various metals and other materials such as nickel oxide, iridium oxide, ruthenium oxide, cobalt phosphate, cobalt oxide and so forth, containing metals with variable valence states, are usually used as cocatalysts [74,77,108,109,110,111,112,113,114] (Table 2).

**Figure 6:**
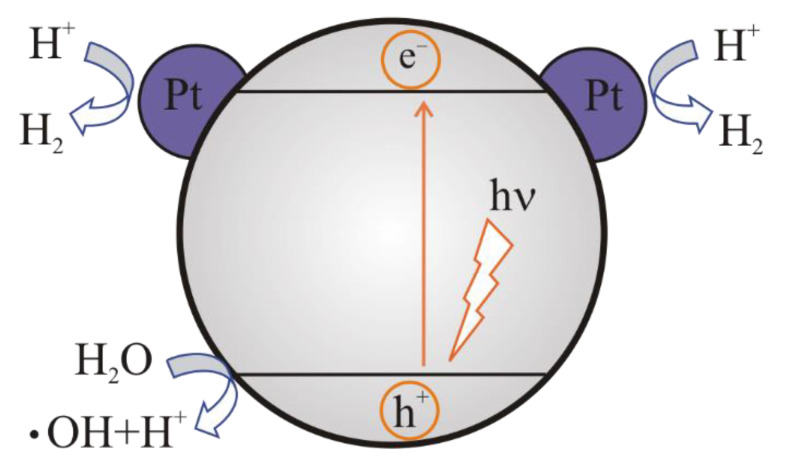
Hydrogen evolution by water splitting over an Pt/HS-TiO_2_ photocatalyst [11].

If the metal particles are deposited on the surface of the photocatalyst (Figure 6), then the Schottky barrier is formed and photoexcited electrons migrate from the semiconductor to the surface since the Fermi level in the metal is lower. The higher the Schottky barrier, the lower the recombination rate between the electron transferred to the metal and the hole, and the greater the production of H_2_ [16]. Electrons are more easily captured by noble metals with higher work functions, so platinum is the best electron-trapping cocatalyst. Photoexcited holes remain in the semiconductor and migrate to its surface, which helps to reduce the probability of a reverse reaction between hydrogen and oxygen, and therefore the yield of a chemical reaction increases. The addition of gold particles to titanate, niobate, and tantalate photocatalysts was found to increase their activity with respect to water decomposition [6].

The photocatalytic performance of a semiconductor can be significantly improved using water oxidation electrocatalysts decreasing the activation energy (*E*_a_) of the process [41]. Taking into account domination of the activation energy of water oxidation over the entire water splitting reaction, it can be concluded that it is necessary to find more efficient H_2_O oxidation cocatalysts for more efficient H_2_ production.

Using the cocatalysts lowers the overvoltage of PEC water splitting, i.e., increases the photocurrent density and ensures a negative shift of onset potential.

Optimal catalyst loading to achieve maximum efficiency in terms of H_2_ evolution rate under visible-light exposure is determined by a balance of two factors. On the one hand, according to [20], the higher the catalyst loading, the higher the H_2_ yield, since the surface area available for the photocatalytic water-splitting reaction increases. On the other hand, catalyst loading should not prevent uniform light penetration over the catalyst surface by reducing the total surface area exposed to light irradiation [115].

## 5. Common Factors Affecting the Efficiency of Photocatalytic Water Splitting

In addition to the high requirements for the photocatalyst material, there are general factors affecting the efficiency of hydrogen production in the process of water splitting.

### 5.1. Irradiation Intensity

Solar illumination for photocatalytic water splitting is usually produced by a standard sunlight simulator with a 300 W xenon lamp. The waveform of the lamp can be reformed as artificial sunlight by an air mass of 1.5 Global (AM 1.5 G) filter with an incident energy density of 100 mW cm^−2^ [3]. The AM 1.5 number representing the spectrum at mid-latitudes is much more common value, since many of the world’s solar industry centers across Europe, China, Japan, the United States of America and elsewhere lie in temperate latitudes.

There are two regimes regarding the photocatalytic reaction with respect to the photon flux. The first-order regime is realized for low-enough fluxes of about 25 mW cm^−2^ at the sample surface [11] when the electron–hole pairs are consumed faster by chemical reactions than by recombination reactions. If the rate of recombination prevails then the half-order regime is realized, resulting in less effect on the rate of reaction. For greater flux intensity in [116], an increase in the photoactivity of ZnS by 20% was recorded due to an increase in the illumination power from 90 to 100 mW cm^−2^. This improvement is not repeated by increasing the light intensity to 110 mW cm^−2^. According to [116], diffusion limitation and rapid recombination black out the effect of light-intensity enhancement.

The influence of the wavelength repeats the adsorption spectrum of the photocatalyst with a threshold corresponding to the band energy [117].

### 5.2. pH of the Solution

The production of H_2_ from water depends on the concentration of protons, i.e., pH of the solution. This aspect is especially important in the presence of a sacrificial reagent. In acidic solution, more H^+^ ions are adsorbed on the surface, so the rate of the reaction (2) increases. However, in general conclusion, based on the previous research, hydrogen production is more efficient in weak alkaline solutions than in acidic or strongly alkaline ones (pH > 10). Moreover, the pH level can influence the corrosion stability of photocatalyst. The maximum efficiency of hydrogen production on the CuO_x_/TiO_2_ heterostructure was registered in a slightly alkaline environment (pH 10) [118], while the minimum efficiency was obtained at pH 2, since Cu(I) has a low stability on the surface of TiO_2_ in an acidic environment. A change in pH can also lead to a modification of the bandgap of the catalyst.

### 5.3. Temperature

There is no direct contribution of temperature to the generation of charge carriers [11], but products’ desorption from the catalyst surface is intensified at elevated temperatures. As a result, the photocatalytic activity of a semiconductor increases. Moreover, at relatively high temperatures the transfer of electrons in VB to higher energy levels is promoted. Therefore, an electron–hole pair formation is facilitated, while their recombination is slowed down [117].

Temperature also increases hydrogen desorption and accelerates the diffusion of sacrificial reagent on the surface of catalyst [34]. In addition, increasing the temperature by one can decrease the Gibbs free energy of water splitting, making it faster and ensuring less energy consumption for thermodynamically improved hydrogen production [119].

The temperature applied differs for different catalyst. Therefore, this factor could be adjusted to increase the photocatalytic activity [11]. For instance, the hydrogen generation was found to increase evolution from 3 folds higher with the Pt/TiO_2_ photocatalyst when the temperature increased from 45 °C to 55 °C [120]. For the WO_3_/FTO photoanode a 10% rise of photocurrent density was revealed at temperature change from 25 °C to 45 °C. Maximal photocurrent of 0.94 mA cm^−2^ was reached at 1.23 V (SHE) for the WO_3_/W photoanode at 25 °C with 29% increasing at 45 °C [121].

The higher temperature does not always result in a hydrogen production increase. It is necessary to perform an optimal temperature interval for the investigated system. High-enough temperatures accelerate the bulk recombination, which slows down the photocatalytic water splitting.

### 5.4. Photocatalyst Dosage

The effect of the amount of photocatalyst loaded into the reactor was investigated, for example, in [23]. It was shown that more TiO_2_ NPs/Ti_3_AlC_2_ composite catalyst loading resulted in higher hydrogen generation, obviously due to larger exposed surface area available for photocatalytic water-splitting reaction. However, hydrogen production per unit weight of catalyst was decreased with more catalyst dosage. Considering photocatalytic activity per unit mass, the photocatalyst can be irradiated by the maximum amount of light for the effective excitation of electrons. Higher dosage of catalyst reduced the total surface area exposed to light irradiation and decreased uniform light penetration over the catalyst surface. Thus, the charge-carrier recombination center per unit mass retards the separation of electron–hole pairs compared to the lower amount of catalyst loading. This reveals that optimum catalyst loading is beneficial in achieving higher efficiency for photocatalytic water splitting under visible-light irradiation.

### 5.5. Surface Effects

The photocatalytic water splitting as a surface chemical reaction involves adsorption of reactive molecules, reaction between photogenerated electrons and holes, adsorbed molecules mediated by photocatalyst or cocatalyst and desorption of product molecules [6]. The reaction efficiency is strongly affected by the quantity and characteristics of the reaction sites. The number of active sites is determined by cocatalyst and water-accessible surface area. The larger surface area will provide more reactive sites, resulting in increased photocatalytic activity [4].

From this point of view, mesoporous structure, 1D nanostructure and 3D hierarchical structures can greatly increase the surface area of the photocatalyst and therefore enhance the hydrogen-generation efficiency.

In accordance with [34], the photocatalytic hydrogen generation from water includes mass transfer. The latter strongly depends on the surface area and porosity of the photocatalyst. The increase in surface area and porosity offers active sites for the water-sacrificial agent to desorb and offer more channels for the process of internal diffusion. That is why dye-sensitized metal–organic frameworks (MOF) with a porous structure and high specific area are assumed to be an effective alternative of high-cost and toxic metals and noble-metal-incorporated MOFs [34]. The organic-dye molecules dispersed on the MOF catalysts are capable of visible-light absorption, leading to efficient charge transfer and hydrogen generation.

Photoconversion efficiency and maximum photocurrent were measured in [16] for TiO_2_ nanotube-array photoanode as a function of the geometric roughness factor. The nanotubular structure improved the charge separation and the electron lifetimes.

Surface recombination at trapping states is another important factor affecting water reduction [6]. The surface states can act as recombination centers [28]. Accordingly, photoexcited charge carriers can be captured in surface states and are available for transfer across the interface. Unfortunately, it is very difficult to obtain direct experimental information about surface states.

## 6. Conclusions

Metal oxide materials can play a significant role in the photocatalytic production of hydrogen from water and sunlight, which is a promising direction in the transition to renewable and carbon-free energy sources. Due to such characteristics as earth abundance, large bandgap, stability in aqueous media, low cost, possibility to vary optoelectronic properties in a wide range and ease to obtain (including electrochemical methods), oxide semiconductors are considered as promising materials for photocatalytic water splitting and good candidates for long-term hydrogen production.

However, the optimum oxide photocatalyst with the properties required for efficient water splitting can be reached only using the catalyst modification. Combining different modification routes makes it possible to formulate the possible ways of synthesis of a semiconductor material with properties required for the most effective light absorption of a wide spectrum, efficient charge transfer and reduced possibility of charge-carrier recombination, high corrosion resistance and photochemical stability, and low cost.

The disadvantage of most oxide semiconductors due to the inappropriate levels of the valence and conduction-band edges can be solved by doping with metal or nonmetal ions or combining with another semiconductor to achieve an optimum band structure and expand the region of the light absorption. The most optimal photoelectrode material characterized by a high efficiency of photocatalytic water splitting can be found using complex three-component and ternary metal oxide-semiconductor materials. The formation of solid solution consisting of wide- and narrow-bandgap oxide and nonoxide semiconductors is a less appropriate method for optimizing the photocatalyst band structure as compared with creation of heterojunctions, combining the properties of different components to increase overall water-splitting efficiency.

It is necessary to find a compromise between an increase in the efficiency of photon absorption and a not-too-high intensification of the recombination of photoexcited charge carriers. The limitations in efficiency of oxide-semiconductor photocatalytic materials because of the fast recombination of electrons and holes can be solved by modifying the crystal structure and morphology of the photocatalyst. The recombination of donor–acceptor pairs can be significantly reduced by the combination of annealing and hydrogen treatment. The morphology and crystallinity of oxide materials can be monitored and regulated, which is most importantly, in the course of their formation. The higher the defectiveness of the oxide material, the higher the efficiency of the water-splitting reaction.

Nanostructuring the oxide semiconductor makes it possible to form material with decreased recombination rates and rather high redox-splitting reaction rate. The negative effect of charge-recombination acceleration because of the developed transport network in nanoparticles, nanowires, nanotubes, nanorods, nanosheets or hierarchical nanostructures can be leveled using heterojunctions due to their synergistic effect upon visible-light irradiation. The prospects for nanostructuring of metal oxide materials to improve the photocatalytic water splitting are determined by comparing them in terms of efficiency; it is necessary to analyze the combined action of several different modification methods, and to identify the multiplicative effect.

Absorption in the visible range can be improved by sensitization of metal oxides with quantum dots, noticeably increasing the photocurrent density. The sequential chemical/electrochemical deposition at the quantum dot/metal oxide interface is a more promising way to extend the light-absorption spectrum and improve charge-carrier separation.

Various metals and other materials containing metals with variable valence states can be used as cocatalysts ensuring formation of active sites for hydrogen evolution and preventing the photoexcited charge-carrier recombination. The main problem here is to find more efficient water molecule oxidation cocatalysts, effectively lowering the overvoltage of photoelectrochemical water splitting, and increasing the photocurrent density, because this reaction dominates in activation energy over the entire water-splitting reaction. An additional possibility to increase the efficiency of oxide materials in hydrogen production relates to the appropriate choice of sacrificial agent. To maximize the efficiency of photocatalytic water splitting, it is advisable to combine the known strategies.

Furthermore, the regime and conditions of the process must be tuned, including light intensity, pH, temperature, applied potential for PEC water splitting and semiconductor material surface area. In order to facilitate the hydrogen production, it is necessary to optimize all factors influencing the rate and the efficiency of both partial processes in overall reaction of photocatalytic and PEC water splitting.

## Figures and Tables

**Table 1:** The efficiency of some sacrificial reagents in hydrogen production.

Year, Ref.	Photo-Catalysts	Bandgap, eV	Light Irradiation Parameters	Reagents	Sacrificial Reagent	Hydrogen Production, mmol g^−1^ h^−1^
2014, [17]	Au/TiO_2_	2.77–3.26	Set of 3 Solarium Philips HB175 lamps each equipped by 4 15 W Philips CLEO florescent tubes	1 g L^−1^ photocatalyst 25 vol.% methanol in water pH ~5	Methanol	0.303–1.543
2015, [18]	Au/TiO_2_	3.03–3.33	Spectroline model SB-100P/F lamp 100 W 365 nm	10 vol.% sacrificial reagents	Glycerol	1.9–27.9
					Ethylene glycol	1.4–20.9
					Methanol	0.9–13.5
					Ethanol	0.4–9.8
2017, [19]	Zn0.5 Cd0.5S g-C_3_N_4_ TiO_2_	-	300 W Xe lamp, wavelength ≥ 420 nm	Aqueous solution 0.2 g L^−1^ photocatalyst powder + 20 vol.% sacrificial reagents	Triethanol amine	1.197
					Formic acid	0.845
					Methanol	0.599
					Methyl amine	0.279
					Ethylene glycol	0.116
					Ethanol	0.111
					Ethylamine	0.101
					Ethylene diamine	0.084
2020, [20]	Cu/In_2_O_3_ /TiO_2_ NPs Cu/In_2_O_3_ NRs /TiO_2_ NWs	2.69 2.90	35 W HID lamp, light intensity 20 mW cm^−2^, wavelength 450 nm	0.01 g of photocatalyst was dispersed in 130 mL aqueous solution + 10 vol.% sacrificial reagent	Glycerol	6.09
					Ethylene glycol	4.85
					Methanol	4.39
					Ethanol	2.84
2020, [21]	TiO_2_ NPs TiO_2_ MPs	3.20 3.10	35 W HID Xenon lamp, 20 mW cm^−2^, wavelength ~420 nm	0.1 g of photocatalyst catalyst was dispersed in 100 mL water containing sacrificial reagent	Glycerol	9.073
					Methanol	4.574
					Phenol	0.146
					0.2 M Na_2_S/Na_2_SO_3_	0.508
					0.1 M Na_2_S/Na_2_SO_3_	0.124

**Table 2:** Photocurrent density in systems with different cocatalysts.

Year, Ref.	Catalysts	Bandgap, eV	Light Irradiation Parameters	Solution	Cocatalyst	Photocurrent Density, mA cm^−2^
2012, [74]	Cu/nano Cu_2_O	2.0	LED light illumination 26 mW cm^−2^ λ = 425–660 nm	0.1 M Na_2_SO_4_ pH = 6	-	–0.140
					NiOx	–0.415
2018, [108]	BiVO_4_	2.4	500 W Xe arc lamp, 100 mW cm^−2^ AM 1.5 G	0.25 M K_2_B_4_O_7_ + 0.2 M Na_2_SO_4_ pH = 9.5	-	~1
					Fe_2_TiO_5_	3.23
2019, [109]	CdS	-	100 mW cm^−2^ AM 1.5 G	0.1 M Na_2_SO_4_ + 0.1 M Na_2_SO_3_ + 0.01 M Na_2_S	-	3.1
					MoS_2_	4.8
					MoSC	7.7
2019, [110]	CuInS_2_ /Sb_2_S_3_	~1.5	100 W Xe lamp, 100 mW cm^−2^ AM 1.5 G	0.1 M Na_2_SO_4_	-	–1.86
					Pt	–2.48
2020, [77]	FTO/CuO	1.5	Xe lamp 100 mW cm^−2^ AM 1.5 G	0.5 M Na_2_SO_4_ (pH = 6)	-	–0.92
					NiO	–1.02
2021, [111]	BiVO_4_	2.45	150 W Xe lamp, 100 mW cm^−2^ AM 1.5 G	0.1 M PBS solution (pH = 7)	-	0.03
					CoOOH	1.10
2021, [112]	ZnIn_2_S_4_	2.4–2.8	AM 1.5 G illumination	0.2 M Na_2_SO_4_	-	0.12
					Mg^2+^	0.38
					Co^2+^	0.54
					Co^2+^|Mg^2+^	0.92
2021, [113]	Ge_3_N_4_	3.4	300 W Hg lamp	0.5 M Na_2_SO_4_	-	2.9
					Mo_2_N	3.7
					CoO_x_	4.1
					CoO_x_Mo_2_N	5.6
					CoO_x_-Mo_2_N	9.2
2022, [114]	NiFeOOH/BiVO_4_	2.41	Xe lamp, 100 mW cm^−2^ AM 1.5 G	0.5 M Na_2_SO_4_ pH = 7.35	-	1.9
					Co–Sil *	2.1
					Co–Pi *	2.2
					Co–Ci *	4.1

* Cobalt−silicate, cobalt−phosphate and cobalt−carbonate cocatalysts are signed in the Table 2 as Co–Sil, Co–Pi and Co–Ci
